# Clinical manifestations of *Mycobacterium abscessus* lung disease according to different morphotypes

**DOI:** 10.7189/jogh.16.04128

**Published:** 2026-05-22

**Authors:** Ling-Kai Chang, Yu-Feng Wei, Ping-Huai Wang, Sheng-Wei Pan, Chin-Chung Shu, Hao-Chien Wang, Chong-Jen Yu

**Affiliations:** 1 Department of Medicine, National Taiwan University Cancer Center, Taipei, Taiwan; 2 College of Medicine, National Taiwan University, Taipei, Taiwan; 3 Department of Internal Medicine, E-Da Cancer Hospital, Kaohsiung, Taiwan; 4 School of Medicine for International Students, I-Shou University, College of Medicine, Kaohsiung, Taiwan; 5 Department of Thoracic Medicine, Far Eastern Memorial Hospital, New Taipei City, Taiwan; 6 School of Medicine, National Yang Ming Chiao Tung University, Taipei, Taiwan; 7 Department of Nursing, Asia Eastern University of Science and Technology, New Taipei City, Taiwan; 8 Department of Chest Medicine, Taipei Veterans General Hospital, Taipei, Taiwan; 9 Department of Internal Medicine, National Taiwan University Hospital, Taipei, Taiwan; 10 Graduate Institute of Clinical Medicine, National Taiwan University College of Medicine, Taipei, Taiwan

## Abstract

**Background:**

*Mycobacterium abscessus* complex (MABC) displays phenotypic heterogeneity, primarily characterised by two distinct morphotypes: smooth and rough. However, the clinical manifestations, radiographic findings, and treatment outcomes of the two MABC morphotypes remain insufficiently characterised and poorly understood. We aimed to investigate the association between these morphotypes and clinical manifestations in a Taiwanese MABC-lung disease cohort.

**Methods:**

We conducted this study at a single centre in northern Taiwan. We enrolled patients diagnosed with MABC-lung disease (LD) and analysed their clinical characteristics in relation to MABC morphotypes. We also evaluated radiographic findings.

**Results:**

Among 121 patients with MABC-LD, the MABC isolates of 80 (66.1%) patients were smooth variants, and the remaining 41 (33.9%) were rough variants. Fewer patients with the rough variant of MABC had COPD and were smokers compared to patients with the smooth variant, but there were more patients with the rough variant (22.0%) compared to those with the smooth variant (5.0%) showing a fibrocavitary (FC) pattern on chest imaging (*P* = 0.017). Multivariable logistic regression analysis identified the rough morphotype as an independent factor associated with the FC pattern (adjusted odds ratio (aOR) = 4.741; 95% CI = 1.403–18.870, *P* = 0.016). Conversely, the FC pattern was the only significant correlated factor of rough morphotype (OR = 4.196; 95% CI = 1.183–17.771, *P* = 0.033).

**Conclusions:**

MABC-LD patients with rough-variant isolates had a higher rate of FC pattern compared with those with smooth-variant isolates. Given that an FC pattern may indicate poor prognosis, morphotypes of clinical isolates in MABC-LD may serve as a potential marker to guide clinical management, although further validation is needed.

Infections caused by nontuberculous mycobacteria (NTM) are increasing worldwide. *Mycobacterium abscessus* complex (MABC), a rapidly growing *mycobacterium*, is responsible for both pulmonary and extrapulmonary diseases in humans [1–4]. Diagnosing MABC-lung disease (MABC-LD) is particularly challenging, as it requires a combination of clinical symptoms, radiographic findings, and microbiological evidence [5–7].

Among patients with MABC-LD, *M. abscessus* exhibits two distinct macroscopic morphotypes: the smooth and the rough variants. The smooth variant expresses glycopeptidolipids (GPLs) on its surface, which facilitate immune evasion, whereas the rough variant lacks them [8,9]. In rough variants of MABC, the loss of GPL exposes immunostimulatory toll-like receptor-2 agonists, such as lipoproteins, thereby triggering an exaggerated pro-inflammatory response that contributes to acute and severe infections [9]. In mouse studies, the rough variant demonstrated greater pathogenicity, with prolonged lung persistence and dissemination to the spleen, whereas the smooth variant was efficiently cleared from the lungs within three weeks [10]. Another study also demonstrated that cytokine production by human peripheral blood mononuclear cells and monocytes differs between MABC variants. Monocytes exhibited a markedly reduced ability to internalise rough variants compared to smooth variants. This may be attributed to the larger size of rough variants, making them more difficult to phagocytose [11]. Additionally, rough MABC strains induced a distinct cytokine profile compared to smooth strains, suggesting that colony morphology plays a key role in host-pathogen interactions. Rough variants elicited significantly higher production of the pro-inflammatory cytokine IL-1β, whereas IL-6 and IL-8 were produced in comparable amounts by peripheral blood mononuclear cells stimulated with either smooth or rough MABC variants [12]. Moreover, the rough variant forms serpentine cords, potentially amplifying pro-inflammatory signalling and promoting apoptosis [1,12–14].

Notably, although the immune response differs between MABC morphotypes, the clinical impact of the two MABC variants remains unclear, as only a limited number of studies have addressed this issue. Hedin and colleagues [15] reported a series of 40 MABC-LD cases, 15 of which involved the rough variant. The rough variant was associated with a lower likelihood of clinical cure, a higher cough frequency, and more extensive cavitary lesions. Li and colleagues [16] analysed 182 cases of MABC-LD, 159 of which involved the rough variant. Findings revealed that patients infected with the rough variant exhibited higher levels of inflammatory markers, poorer lung function, more cavitary lesions, and a higher rate of disease exacerbation [14,16].

On the other hand, clinical and radiological progression is a key concern in MABC-LD [17,18]. To date, large cohort studies systematically addressing this issue are lacking. In our earlier work, we found that persistent acid-fast stain (AFS) positivity and higher AFS grades were associated with an increased risk of disease progression in MABC-LD [6]. However, the potential contribution of the bacteria morphotypes in MABC-LD has yet to be elucidated, and its clinical impact remains unclear. Therefore, we aimed to further investigate this association in a Taiwanese MABC-LD cohort.

## METHODS

### Design, setting, and patients

We conducted this study at a single medical centre in northern Taiwan – the National Taiwan University Hospital. Owing to its retrospective design, the requirement for written informed consent was waived. Between January 2011 and December 2019, we identified patients through the microbiological database based on the presence of at least two separate MABC-positive sputum cultures within a 12-month period. We retrospectively reviewed medical records, chest radiographic images, and follow-up microbiological data. We excluded patients who had disseminated MABC infection, concurrent *M. tuberculosis* or other NTM infections at diagnosis, isolated MABC colonisation, no microbiological follow-up beyond the first year, or were HIV-positive.

### Definition and measurements

Mycobacterial cultures were conducted at National Taiwan University Hospital, and MABC species were identified using conventional biochemical methods [19]. Patients with at least two MABC-positive sputum samples underwent evaluation for MABC-LD based on the diagnostic criteria established by the American Thoracic Society [20]. Those who tested positive for MABC in at least two sputum samples but did not meet the American Thoracic Society diagnostic criteria were categorised as having MABC colonisation and excluded from the study. For eligible patients, we set the index date to the date of the first MABC-positive sputum sample.

The smooth and rough variant of MABC is defined by the colony morphology on the solid culture medium of 7H11 [15]. MABC subspecies was identified by analysing gene sequences of secA1, rpoB, and hsp65 using polymerase chain reaction, followed by direct sequencing [21]. Based on the genetic sequence analysis, the variants were classified into *M. abscessus* subsp *abscessus* or *M. abscessus* subsp *massiliense*.

We conducted a review of medical records, systematically coding key clinical parameters, including age, sex, body mass index (BMI), presenting symptoms, comorbidities, radiographic findings, AFS grading of sputum samples, mycobacterial culture results, and MABC-LD treatment. Pulmonologists evaluated chest radiographs and, when available, concurrently analysed computed tomography scans. Pulmonologists, blinded to all clinical and microbiological data (including bacterial morphotypes), performed radiographic scoring according to the criteria Snider and colleagues [22] established (Figure S1 in the **Online Supplementary Document**). They categorised radiographic patterns into nodular bronchiectatic, fibrocavitary (FC), or other types based on established classification criteria [23,24]. We provided the evaluators solely with anonymised case identifiers and the corresponding imaging studies to ensure an objective interpretation of the radiographic patterns.

Our primary endpoint was the FC pattern, and the secondary endpoint was the clinical disease progression. We defined clinical progression as symptomatic worsening (*e.g.* cough, sputum production, or weight loss) or radiographic deterioration (*e.g.* increased pulmonary involvement or the emergence of new cavities or nodules) necessitating the initiation of guideline-based antibiotic therapy for MABC-LD.

### Statistical analysis

We analysed continuous variables using Student’s *t* test or the Mann-Whitney U test, depending on the data distribution. We summarised categorical variables as counts and percentages and conducted group comparisons using either the χ^2^ test or Fisher exact test, depending on the data distribution.

To identify factors associated with a rough variant MABC-LD or FC pattern, we performed logistic regression, calculating odds ratios (ORs) and adjusted ORs (aORs) with 95% confidence intervals (CIs) and *P*-values. For the multivariable regression models, we included clinically relevant variables (eg, age, sex, and BMI) as well as those that showed a potential association (*P* < 0.05) in the univariate analysis. We used Cox proportional hazard regression to analyse the effect of MABC morphotype on clinical progression.

We used *R*, version 4.0.3 (R Core Team, Vienna, Austria) for all analyses.

## RESULTS

### Patient enrolment and demographics

**Figure 1:**
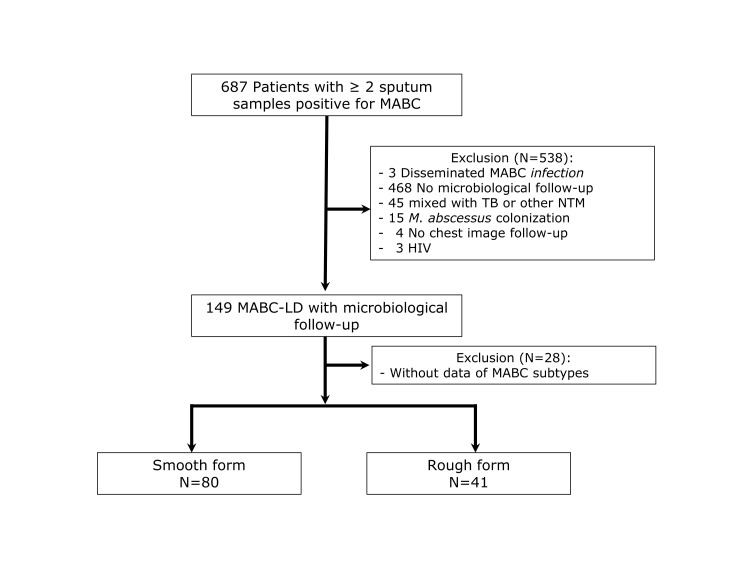
Flowchart of patient enrollment.

During the study period, a total of 687 patients with at least two MABC-positive sputum cultures within a 12-month period were identified at the study hospital. We applied a stepwise exclusion process for the following reasons: disseminated MABC disease (n = 3), lack of microbiological follow-up (n = 468), concurrent tuberculosis or other NTM infections (n = 45), MABC colonisation (n = 15), absence of follow-up chest imaging (n = 4), HIV infection (n = 3), and missing subspecies identification data (n = 28) (**Figure 1**). After applying these criteria, we included 121 patients with a confirmed diagnosis of MABC-LD in the final analysis (**Table 1**).

**Table 1:** Clinical characteristics of the patients*

	Total (n = 121)	Smooth group (n = 80)	Rough group (n = 41)	*P*-value
**Age in years, x̄ (SD)**	62.5 (15.4)	63.6 (14.8)	60.4 (16.4)	0.290
**Female sex**	46 (38.0)	32 (40.0)	14 (34.1)	0.716
**BMI, x̄ (SD)**	21.0 (4.0)	21.0 (4.1)	21.1 (3.7)	0.868
**Smoking**				0.011
Never smoker	105 (86.8)	65 (81.3)	40 (97.6)	
Current/former smoker	16 (13.2)	15 (18.7)	1 (2.4)	
**Cavitary**	10 (8.3)	4 (5.0)	6 (14.6)	0.166
**Follow-up time in years, MD (IQR)**	4.1 (2.9–5.2)	4.1 (2.9–5.1)	3.6 (2.9–5.3)	0.369
**Symptoms**				
Cough	85 (70.2)	54 (67.5)	31 (75.6)	0.767
Shortness of breath	28 (23.1)	22 (27.5)	6 (14.6)	0.261
Haemoptysis	40 (33.1)	26 (32.5)	14 (34.1)	>0.999
**Comorbid condition**				
Cancer	26 (21.5)	19 (23.8)	7 (17.1)	0.646
Diabetes mellitus	15 (12.4)	12 (15.0)	3 (7.3)	0.385
COPD	15 (12.4)	14 (17.5)	1 (2.4)	0.037
History of TB	16 (13.2)	9 (11.2)	7 (17.1)	0.578
**Radiographic pattern**				
NB	96 (79.3)	67 (83.8)	29 (70.7)	0.103
FC	13 (10.7)	4 (5.0)	9 (22.0)	0.017
**Radiographic score, MD (IQR)**	6 (3–9)	5 (3–8)	7 (6–9)	0.022
**Highest AFS grade in initial year, MD (IQR)**	1 (0–3)	0.5 (0–2)	1 (0–3)	0.077
**Initial year AFS positivity**				0.206
0	51 (42.1)	38 (31.4)	13 (31.7)	
0.5–2	36 (29.8)	23 (19.0)	13 (31.7)	
3–4	34 (28.1)	19 (15.7)	15 (36.6)	
**Transient anti-TB medication (≥1 mo)**	16 (13.2)	11 (13.8)	5 (12.2)	>0.999
**MABC clinical progression**	70 (57.9)	44 (55.0)	26 (63.4)	0.753
**MABC radiographic progression**	57 (47.1)	36 (45.0)	21 (51.2)	0.648
**Subspecies with *M. abscessus* subsp *abscessus***	64 (52.9)	44 (55.0)	20 (48.8)	0.771
**NTM treated within 1 y of diagnosis**				0.550
No	107 (88.4)	72 (90.0)	35 (85.4)	
Yes	14 (11.6)	8 (10.0)	6 (14.6)	
**NTM which had been treated**				0.847
No	70 (57.9)	47 (58.8)	23 (56.1)	
Yes	51 (42.1)	33 (41.2)	18 (43.9)	

AFS – acid-fast stain, BMI – body mass index, COPD – chronic obstructive pulmonary disease, FC – fibrocavitary, IQR – interquartile range, MABC – *mycobacterium* *abscessus* complex, MD – median, NB – nodular bronchiectatic, SD – standard deviation, TB – tuberculosis, x̄ – mean

*Values are presented as numbers (percentages) unless specified otherwise.

### Clinical characteristics of patients

Among the 121 enrolled patients with MABC-LD (**Table 1**), 80 (66.1%) had the smooth variant (smooth group), and 41 (33.9%) had the rough variant (rough group). The mean (x̄) age of the overall cohort was 62.5 years, with no significant difference between the smooth (x̄ = 63.6) and rough (x̄ = 60.4) groups (*P* = 0.290). Female patients accounted for 38.0% of the overall cohort, with no difference in the proportion between the smooth (40%) and rough (34%) groups (*P* = 0.716). The average BMI also did not differ between the smooth (x̄ = 21.0) and rough (x̄ = 21.1) groups (*P* = 0.868).

A significantly higher proportion of patients in the smooth group (18.7%) than those in the rough group (2.4%) had a history of smoking (*P* = 0.011). The median (MD) follow-up duration was 4.1 years (IQR = 2.9–5.2), with no significant difference between the two groups (*P* = 0.369). Cough was the most reported symptom, affecting 70.2% of patients. Shortness of breath (23.1%) and haemoptysis (33.1%) were also frequent within the cohort, with no significant differences between the smooth and rough groups (*P* > 0.05). The most prevalent comorbidity was cancer (21.5%), followed by diabetes mellitus (12.4%) and chronic obstructive pulmonary disease (COPD) (12.4%). Notably, COPD was significantly more common in the smooth (17.5%) than in the rough (2.4%) group (*P* = 0.037). A history of tuberculosis was identified in 13.2% of patients, with no significant difference between groups (*P* = 0.578).

### Radiographic findings

Most patients (79.3%) exhibited a nodular bronchiectatic pattern (**Table 1**). However, the FC pattern was more frequent in the rough (22.0%) than in the smooth (5.0%) group (*P* = 0.017). Additionally, the radiographic score was significantly higher in the rough (MD = 7; IQR = 6–9) compared to the smooth (MD = 5; IQR = 3–8) group (*P* = 0.022).

### Microbiological characteristics

The highest AFS grade in the first year was higher in the rough (MD = 1; IQR = 0–3) than in the smooth (MD = 0.5; IQR = 0–2) group, although not statistically significant (*P* = 0.077). Regarding AFS positivity in the first year, 42.1% were negative, 29.8% had grades 0.5–2, and 28.1% had grades 3–4, with no significant difference between groups (*P* = 0.206). Transient use of anti-tuberculosis medications for more than one month was observed in 13.2% of patients, with no significant difference between groups (*P* > 0.999). MABC-LD clinical progression occurred in 57.9% of patients, while radiographic progression was observed in 47.1%, with no significant differences between the groups. A higher proportion of patients in the rough group than in the smooth group received NTM treatment within the first year after diagnosis and throughout the entire follow-up period, although neither difference was statistically significant (**Table 1**).

### Subspecies distribution

MABC subsp *abscessus* accounted for 52.9% (**Table 1**). The distribution of subspecies did not significantly differ between the smooth and rough groups (*P* = 0.771).

### Logistic regression analysis of rough-variant MABC-LD

In the univariable analysis using the rough variant as the outcome, several factors were significantly associated with the rough morphotype of MABC-LD (**Table 2**). These included the presence of a FC pattern (OR = 5.344; 95% CI = 1.616–20.900, *P* = 0.008), higher radiographic score (OR = 1.119; 95% CI = 1.004–1.253, *P* = 0.045), COPD (OR = 0.118; 95% CI = 0.006–0.621, *P* = 0.043), and history of smoking (OR = 0.108; 95% CI = 0.006–0.566, *P* = 0.035). However, in the multivariable logistic regression analysis, only the FC pattern remained independently associated with the rough variant (OR = 4.196; 95% CI = 1.183–17.771, *P* = 0.033).

**Table 2:** Univariable and multivariable logistic regression analysis with rough variant as the outcome

	Univariable analysis		Multivariable analysis*	
	**OR (95% CI)**	***P*-value**	**aOR (95% CI)**	***P*-value**
**Age**	0.986 (0.962–1.011)	0.272		
**Female sex**	0.778 (0.349–1.691)	0.531		
**BMI**	1.008 (0.913–1.111)	0.871		
**Smoker**	0.108 (0.006–0.566)	0.035	0.335 (0.016–2.493)	0.356
**COPD**	0.118 (0.006–0.621)	0.043	0.197 (0.009–1.643)	0.183
**FC pattern**	5.344 (1.616–20.900)	0.008	4.196 (1.183–17.771)	0.033
**Radiographic score**	1.119 (1.004–1.253)	0.045	1.095 (0.974–1.235)	0.132
**Highest AFS grade in the initial year**				
0	ref			
0.5–2	1.652 (0.652–4.214)	0.288		
3–4	2.308 (0.920–5.908)	0.076		
**Subspecies with *M. abscessus* subsp *abscessus***	0.750 (0.347–1.617)	0.462		
**Antibiotic used**	1.115 (0.518–2.385)	0.780		
**Duration of disease**	0.994 (0.980–1.008)	0.413		
**Immune status**				
DM	0.447 (0.098–1.514)	0.234		
Cancer	0.661 (0.238–1.673)	0.399		
ESRD	0.359 (0.053–1.449)	0.200		

aOR – adjusted odds ratio, AFS – acid-fast stain, BMI – body mass index, CI – confidence interval, COPD – chronic obstructive pulmonary disease, DM – diabetes mellitus, ESRD – end-stage renal disease, FC – fibrocavitary, OR – odds ratio, ref – reference

*We included all significant factors (*P* < 0.05) from the univariate analysis into the multivariable analysis.

### Logistic regression analysis of FC pattern

In the univariable logistic regression analysis (**Table 3**), rough variant morphology (OR = 5.344; 95% CI = 1.616–20.900, *P* = 0.008) and higher radiographic scores (OR = 1.198; 95% CI = 1.018–1.427, *P* = 0.033) were significantly associated with the presence of a FC pattern. The highest AFS grade within the initial year showed a trend toward significance, particularly for grades 0.5–2 (OR = 4.900; 95% CI = 1.052–34.946, *P* = 0.061) and 3–4 (OR = 4.224; 95% CI = 0.851–30.839, *P* = 0.097), although these were not statistically significant.

**Table 3:** Univariable and multivariable logistic regression analysis with FC pattern as the outcome

	Univariable analysis		Multivariable analysis*	
	**OR (95% CI)**	***P*-value**	**aOR (95% CI)**	***P*-value**
**Age**	0.968(0.934–1.003)	0.067		
**Female sex**	0.698(0.180–2.293)	0.570		
**BMI**	0.838(0.691–0.991)	0.054		
**Smoker**	0.208(0.002–1.709)	0.175		
**COPD**	0.560(0.030–3.194)	0.591		
**Rough form**	5.344(1.616–20.900)	0.008	4.741(1.403–18.870)	0.016
**Radiographic score**	1.198(1.018–1.427)	0.033	1.187(0.990–1.439)	0.068
**Highest AFS grade in the initial year**				
0	ref			
0.5–2	4.900(1.052–34.946)	0.061		
3–4	4.224(0.851–30.839)	0.097		
**Subspecies with *M. abscessus* subsp *abscessus***	0.680 (0.206–2.181)	0.513		
**Antibiotic used**	2.419 (0.756–8.466)	0.143		
**Duration of disease**	1.015 (0.995–1.035)	0.144		
**Immune status**				
DM	0.223 (0.002–1.848)	0.202		
Cancer	1.109 (0.235–3.982)	0.883		
ESRD	0.286 (0.002–2.413)	0.308		

aOR – adjusted odds ratio, AFS – acid-fast stain, BMI – body mass index, CI – confidence interval, COPD – chronic obstructive pulmonary disease, DM – diabetes mellitus, ESRD – end-stage renal disease, FC – fibrocavitary, OR – odds ratio, ref – reference

*We included all significant factors (*P* < 0.05) from the univariate analysis into the multivariable analysis.

In the multivariable logistic regression analysis (**Table 3**), the FC pattern was independently associated with the rough variant (aOR = 4.196; 95% CI = 1.183–17.771, *P* = 0.033). However, the radiographic score was not statistically significant after adjustment (aOR = 1.187; 95% CI = 0.990–1.439, *P* = 0.068).

### Clinical progression analysis

For clinical progression, there was no significant between-group difference (**Table 1**). In the Cox regression model, although the MABC rough form had a trend for progression (hazard ratio = 1.230; 95% CI = 0.755–2.003), there was no statistical significance in univariate analysis (*P* = 0.406) (Table S1 in the **Online Supplementary Document**).

## DISCUSSION

We evaluated a final cohort of 121 patients with confirmed MABC-LD. We classified patients into smooth (66.1%) and rough (33.9%) morphotype groups. While demographic and most clinical characteristics were comparable between the two groups, a history of smoking and the presence of COPD were significantly more common in the smooth group. In contrast, the rough group exhibited a higher prevalence of the FC pattern and significantly elevated radiographic scores. MABC morphotype, however, was not associated with MABC subspecies or clinical progression. Multivariable logistic regression analysis identified a robust co-occurrence between the rough variant and the FC pattern.

Several previous studies have examined the smooth and rough morphotypes in MABC-LD. In a study by Li and colleagues [16], involving 194 patients of which 126 had the rough variant, the rough morphotype was associated with a higher risk of disease exacerbation and poorer baseline characteristics, including worse lung function and elevated inflammatory markers such as C-reactive protein, tumour necrosis factor-α, and interferon-γ. Hedin and colleagues [15] examined 71 patients with MABC, of whom 23 had the rough variant; however, only 40 cases were defined as MABC-LD. They found a higher frequency of cavity formation in patients with the rough variant, consistent with our findings. In contrast, while they reported a lower clinical cure rate among patients with the rough variant, we did not observe this in our analysis. We believe this discrepancy may be attributed to the retrospective nature of both studies, which can introduce variability and bias. Further prospective studies are warranted to validate these findings.

Previous studies have proposed several mechanisms that may explain why the rough variant is associated with more extensive disease, particularly FC changes on radiographic imaging. One such mechanism involves alterations in the bacterial cell wall. In the rough variant, the loss of surface GPLs disrupts normal lipid-mediated interactions, leading to bacterial adhesion. This aggregation leads to the formation of serpentine cords, which may contribute to enhanced virulence and tissue invasion [1,8,25]. The cord formation is also present in *M. tuberculosis* [8]. Some mechanisms stated that the coiled, multi-bacterial aggregates inhibit macrophage phagocytosis, allowing them to evade the immune system [26–29]. Another proposed mechanism is that the rough variant induces a stronger humoral response due to its higher immunogenicity, which may contribute to more severe tissue damage [1,9,30].

We did not find a correlation between smooth and rough variants with clinical progression. A possible explanation is that there was no standardised anti-MABC treatment protocol, so rough-variant MABC-LD patients might receive more intensive treatment. In our cohort, slightly more patients with the rough variant (n/N = 18/41; 43.9%) received anti-MABC therapy than those with the smooth variant (n/N = 33/80; 41.3%), although the difference was not statistically significant given the relatively small sample size.

There was a higher prevalence of smoking and underlying COPD in the smooth group in our cohort (**Table 1**). A previous study showed that MABC grew better in oxidative stress conditions, such as those induced by cigarette smoke, and that the presence of antioxidants reduced the intracellular viability of MABC [31]. Accordingly, we propose that the smooth variant may have a higher propensity for intracellular growth and thus be more prevalent among smokers and patients with COPD. Besides, another study found that electric cigarettes also enhance the growth of MABC, especially the intracellular type [32]. Another study showed that smoking decreased the autophagy function of macrophages, and thus, allows the intracellular type, smooth variant, to survive more easily in smokers [33].

We found that, while subspecies distribution was accounted for, it was not associated with the FC pattern or clinical manifestations. This finding contrasts with some previous literature suggesting that MABC subsp *abscessus* is associated with more severe disease than subsp *massiliense* [34,35]. This discrepancy may be due to the limited number of cases within each subspecies subgroup in our cohort, which may have lacked the power to identify subspecies as a significant effect modifier. Additionally, the clinical course of MABC-LD is multifaceted, often influenced by the timing of treatment initiation and the specific drug combinations used, which may mask the intrinsic virulence differences between subspecies.

We aimed to contribute to a more inclusive global health perspective by addressing a gap in the literature, which has historically been dominated by Western-centric, cystic fibrosis-focused cohorts. By examining a non-cystic fibrosis population with a high prevalence of COPD and smoking, we provided findings that are more representative of many real-world and resource-limited healthcare settings.

Importantly, we demonstrated that colony morphology serves as a meaningful and accessible phenotypic marker which was associated with radiologic severity (FC pattern). We propose that this visual indicator serves as a practical, cost-effective reference for risk stratification, particularly in clinical settings where advanced molecular or imaging resources are limited and phenotypic assessment remains central to clinical decision-making. Taken together, these findings highlight the relevance of routine laboratory markers and suggest a scalable framework for prognostic evaluation applicable across diverse healthcare environments with varying resource availability.

There are several limitations in our study. First, the retrospective design may have led to incomplete clinical coding and non-standardised treatment approaches. Second, we conducted the study at a single centre in northern Taiwan, and thus, the generalisability of the findings requires validation in other regions. Third, we did not investigate the underlying pathogenic mechanisms associated with different MABC morphotypes. Additionally, although diagnostic and treatment guidelines changed over the study period, we consistently applied the 2020 ATS/ERS/ESCMID/IDSA guidelines for the diagnosis of the NTM lung disease. However, treatment initiation is at the clinician's discretion. We relied on the morphotype identified from the initial clinical isolate. Although in vivo morphotype switching is not frequently reported [36], we did not perform longitudinal monitoring of morphotypes throughout the entire clinical course. Therefore, any potential phenotypic transitions occurring during prolonged treatment or disease progression were not captured. Furthermore, while the overall proportion of missing data was low, complete information was unavailable for certain variables, including BMI (n = 7) and subspecies identification (n = 4). Although these missing values were distributed proportionately between the smooth and rough morphotype groups and are unlikely to have introduced systematic bias, their absence represents a limitation in data completeness. Additionally, although we used social insurance status as a proxy for socioeconomic status, the homogeneity of insurance coverage within our cohort prevented meaningful multivariable adjustment for this potential confounder. Moreover, while our use of logistic regression aligns with the methodology in comparable studies, treating clinical progression as a binary, static outcome may limit the assessment of within-subject temporal variation and the handling of censoring inherent in longitudinal data. Since we used baseline clinical data, these associations should be interpreted as correlations rather than confirmed causal pathways. Lastly, we rigorously selected cases with confirmed MABC-LD, excluding those with colonisation, and our strict inclusion criteria requiring microbiology follow-up resulted in a significant reduction in the final analytical cohort (n = 121). Consequently, our findings may primarily represent patients with greater disease severity, potentially limiting the external validity of the results regarding less symptomatic populations.

## CONCLUSIONS

We found that rough-variant MABC-LD is more frequently associated with FC patterns and tends to present with more extensive radiographic involvement, particularly in patients without a history of COPD or smoking. These findings highlight the potential utility of morphotype identification in guiding radiographic severity and clinical risk stratification. Incorporating morphotype analysis into routine diagnostic workflows may help clinicians identify patients at higher risk for structural lung damage. Future prospective studies are needed to validate these observations across diverse populations and to further elucidate the underlying pathogenic mechanisms linking morphotype and disease severity.

## Additional material


Online Supplementary Document
