# Supplementary material for: Clinical manifestations of Mycobacterium abscessus lung disease according to different morphotypes
Source: J Glob Health. 2026 May 22;16:04128. doi: 10.7189/jogh.16.04128 (PMC13196491; doi:10.7189/jogh.16.04128)
Supplement: Online Supplementary Document [file jogh-16-04128-s001.pdf]

Supplement to: Chang LK, Wei YF, Wang PH, Pan SW, Shu CC, Wang HC, Yu CJ. Clinical manifestations of Mycobacterium abscessus lung disease according to different morphotypes. J Glob Health. 2026;16:04128.

Figure S1. Example of the six lung zones of chest radiographic score

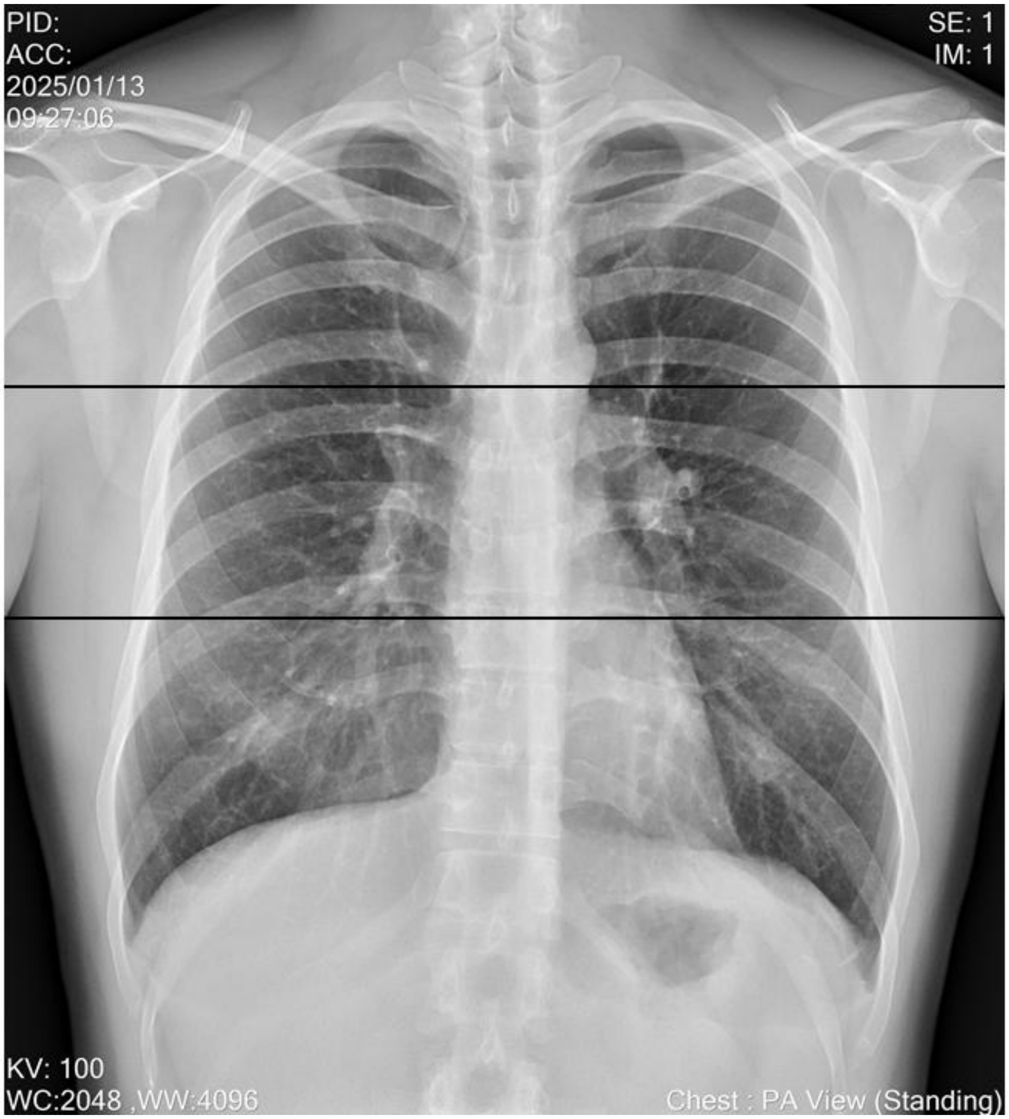

The radiographic score was scored according to Snider et al. study (**Ref.**). We divided the lung field into six zones according to two horizontal lines located at the distal end of the lobar pulmonary artery (Figure E1 as below). Then, we calculated the score from 0 to 3 points at each lung zone. If the lesion is about equal to or less than one-third of the area, it was coded as one point. If a lesion involved more than one-third but less

than two-thirds of the area, two points were coded. If a lesion involved more than two-thirds of the lung zone, three points were coded.

Ref: Snider GL, Doctor L, Demas TA, Shaw AR. Obstructive airway disease in patients with treated pulmonary tuberculosis. Am Rev Respir Dis. 1971;103:625–640.

Table S1. Univariate and multivariable Cox regression analysis of MABC-LD clinical progression

Characteristic	Number of patients	Univariate HR (95% CI)	*P* value	Multivariable HR (95% CI)	*P* value
Age ≧65 vs. <65, years	57/64	1.070 (0.669, 1.710)	0.778		
Female sex	46/75	0.812 (0.490, 1.346)	0.419		
BMI ≧18.5 vs. <18.5	87/27	0.564 (0.326, 0.973)	0.040	0.707 (0.400, 1.252)	0.234
Morphotype: Rough vs Smooth	41/80	1.230 (0.755, 2.003)	0.406		
Radiological score		1.091 (1.025, 1.161)	0.006	1.056 (0.977, 1.141)	0.170
Highest AFS in the initial year					
0	51	reference		reference	
0.5-2	36	2.816 (1.525, 5.201)	<0.001	2.094 (1.103, 3.975)	0.024
3-4	34	3.729 (2.023, 6.872)	<0.001	3.299 (1.718, 6.338)	<0.001
Persistent positivity of AFS	50/71	2.243 (1.393, 3.611)	0.001	2.081 (1.253, 3.456)	0.005

Abbreviations: AFS, Acid-fast stain; BMI, Body mass index; CI, Confidence interval; MABC, Mycobacterium *abscessus* complex

Text S1. Explanation of authorship change statement

During the manuscript submission process, due to the authors' oversight, the authorship byline did not accurately reflect the relative contributions of the authors. Specifically, authors CCS, HCW, and SWP were initially listed as fourth, fifth and sixth authors, respectively. Following re-evaluation of author contributions, author SWP was moved from the sixth to the fourth position, while CCS and HCW were moved to the fifth and sixth author positions, resulting in the revised order of SWP, CCS, and HCW.

This has now been corrected, and the updated list of authors is as follows: LKC, YFW, PHW, SWP, CCS, HCW, CJY.

The authors regret this omission and any inconvenience it may have caused.

All authors filled out and signed the Journal’s authorship change form, have reviewed and agreed to this correction, and confirm that the requested changes accurately reflect each author’s contributions in accordance with the Journal’s authorship policies.
